# Misalignment of Stakeholder Incentives in the Opioid Crisis

**DOI:** 10.3390/ijerph17207535

**Published:** 2020-10-16

**Authors:** Alireza Boloori, Bengt B. Arnetz, Frederi Viens, Taps Maiti, Judith E. Arnetz

**Affiliations:** 1 Department of Statistics and Probability, Michigan State University, East Lansing, MI 48824, USA; viens@msu.edu (F.V.); maiti@msu.edu (T.M.); 2 Department of Family Medicine, Michigan State University, Grand Rapids, MI 49503, USA; arnetzbe@msu.edu (B.B.A.); arnetzju@msu.edu (J.E.A.)

**Keywords:** opioid crisis, opioid/substance use disorder, pain management, misalignment of incentives, stakeholders, cost, quality, access

## Abstract

The current opioid epidemic has killed more than 446,000 Americans over the past two decades. Despite the magnitude of the crisis, little is known to what degree the misalignment of incentives among stakeholders due to competing interests has contributed to the current situation. In this study, we explore evidence in the literature for the working hypothesis that misalignment rooted in the cost, quality, or access to care can be a significant contributor to the opioid epidemic. The review identified several problems that can contribute to incentive misalignment by compromising the triple aims (cost, quality, and access) in this epidemic. Some of these issues include the inefficacy of conventional payment mechanisms in providing incentives for providers, practice guidelines in pain management that are not easily implementable across different medical specialties, barriers in adopting multi-modal pain management strategies, low capacity of providers/treatments to address opioid/substance use disorders, the complexity of addressing the co-occurrence of chronic pain and opioid use disorders, and patients’ non-adherence to opioid substitution treatments. In discussing these issues, we also shed light on factors that can facilitate the alignment of incentives among stakeholders to effectively address the current crisis.

## 1. Introduction

From 1999 to 2018, the total number of drug-related deaths in the U.S. increased from 16,849 to 67,367. Among them, opioid analgesics have contributed the most, from 8048 to 46,802, a 481.54% increase [1]. This dramatic change has contributed to a substantial drop in the average life expectancy in the U.S. for consecutive years, particularly among men [2,3]. A myriad of factors have been attributed to the opioid crisis. These include:

- Regulations on reimbursement policies, such as the Hospital Consumer Assessment of Healthcare Providers and Systems (HCAHPS) survey, which partially rewards healthcare systems and providers when patients score high on pain management experience of care, thus making providers more inclined towards opioid analgesics [4];
- Lack of a comprehensive multi-modal pain management strategy [5];
- Barriers in adopting treatments for opioid /substance use disorders [6];
- Efforts that have influenced downplaying the negative impacts of opioids, such as marketing by pharmaceutical companies and professional associations promoting opioids [7] or pain being declared as the fifth vital sign [8];
- Regarding the novel coronavirus 2019 (COVID-19) pandemic, we note that second-order effects have been reported on the opioid crisis; e.g., an increase in rates of opioid use disorders [9] and opioid use in intensive care units during mechanical ventilation [10].

To address the opioid crisis, many remediation actions and policies have been implemented. For example, the Centers for Disease Control and Prevention (CDC) have proposed a set of guidelines for prescribing opioids for chronic pain conditions [11], with the main focus being on avoiding such medications to the greatest possible extent. As of 1 October 2019, the Centers for Medicare and Medicaid Services (CMS) removed pain management questions from the HCAHPS survey [12]. Although the pain management measure in the survey accounts for a small portion of Medicare’s reimbursements/incentives paid to hospitals, it may have incentivized providers to prescribe opioid medications [4]. These actions have been part of a concerted effort to curb the prescription of opioids [13]. As reported by the National Institute on Drug Abuse [1], the number of deaths in the U.S. caused by prescription opioids dropped from 17,087 in 2016 to 14,975 in 2018. Despite this decrease, limiting opioid prescriptions may already have had other repercussions, such as a dramatic increase in deaths from synthetic opioids (e.g., fentanyl), from 19,413 to 31,335 during the same period [1].

Based on these premises, we pose that one of the main issues that contributes to the opioid crisis is the absence of a systemic perspective where (dis)incentives of various stakeholders are taken into consideration in promoting patients’ health and safety. It is imperative to understand the incentive misalignment from a system perspective if we are to effectively combat the epidemic. To this end, we pursue the following objectives in this review: (1) we evaluate evidence in the extant literature surrounding factors that can potentially cause misalignment among stakeholders by compromising either cost, quality, or access to care in this epidemic and (2) identify efforts and strategies that may contribute to alleviating such misalignments.

## 2. Methods

We conducted a literature review by addressing and summarizing potential sources of incentives’ misalignment and their impacts on the opioid crisis [14]. In conducting this review, we searched two databases: Medline (PubMed) and Web of Science. For our search mechanism, we identified three main categories based on the type of medication originating the epidemic, the type of problem/objective we attempt to address in this review, and the type of stakeholders involved along with some of their strategies. For these categories, we also used a series of search terms. These categories are represented as follows, respectively:

- Category 1: “opioid”.
- Category 2: “alignment”, “misalignment”, “align”, “misaligned”, and “incentive”.
- Category 3: “stakeholder”, (“societal planner”, “payer”, “insurer”, “insurance”, or “coverage”), (“payment”, “reimbursement”, “fee-for-service”, “capitation”, “capitated”, “pay-for-performance”, “bundled payment”, “accountable care”, or “value-based”), (“provider”, “physician”, or “hospital”), “patient”, (“employer” or “employment”), (“pharmaceutical” or “drug”), (“pharmacologic”, “non-pharmacologic”, or “nonpharmacologic”), (“barrier” or “facilitator”), and (“contingent” or “contingency”).

To search key terms/words in the title, abstract, or main body of studies, we then used a combination of Categories 1–3. For example, one option is the combination of “opioid”, “alignment”, and (“provider” or “physician” or “hospital”). The timeline for our search encompassed studies published (online) between 1 January 2000 and 31 December 2019. Only studies published in English were included. Of note, to account for the sheer number of references in the literature, we limit the number of databases to two, our search timeline, and the number of search terms for Category 1.

## 3. Results

**Figure 1:**
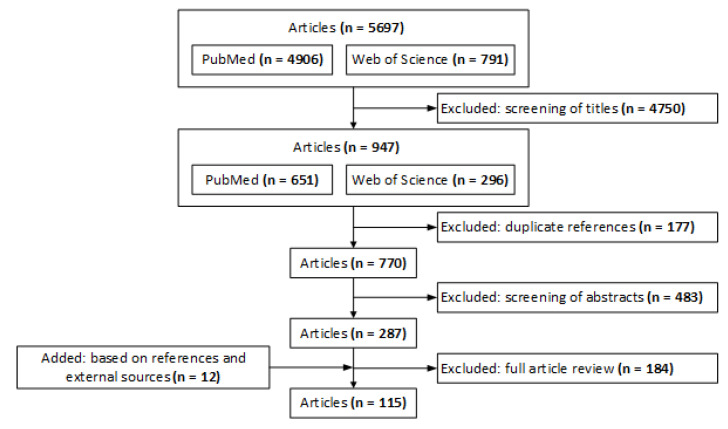
Results of the literature review search method.

From a total of 5697 articles identified, our review resulted in 115 studies (see Figure 1). Among the original 5697 articles that we identified, we sequentially filtered out 4750 by screening of titles, 177 due to duplicate references in our two databases, 483 by screening of abstracts, and 184 by full article review. In reviewing the 115 studies, we noted that factors and dynamics that typically cause misalignment can be dichotomized into two stages, depending on whether or not a patient has been diagnosed with (or is in danger of) opioid/substance use disorder (OUD/SUD). Our review identified five categories of stakeholder misalignment prior to OUD onset and two sources of misalignment subsequent to OUD (Table 1). In Section 3.1 and Section 3.2, we separately discuss the literature for each of these prevention and intervention stages, respectively. In Table 1, we also present a summary of these studies along with types of stakeholders, sources of misalignment addressed, and number and date ranges of studies published under each topic. To this end, we note that the vast majority of papers have been published within 2010–2019 (the second half of our search timeline). In Table 2, we provide the glossary of some terms used commonly throughout the paper.

### 3.1. Stakeholder Misalignment Before Onset of OUD/SUD: A Prevention Perspective

#### 3.1.1. Payment Mechanisms, Reimbursement Schemes, and Incentives

There is evidence that outcomes such as inappropriate prescribing of opioids and diagnosed opioid misuse are more prevalent among fee-for-service (FFS) enrollees compared to patients with other health insurance plans like managed care [15,16]. This is in part due to the fact that payment mechanisms, such as FFS and capitation, would not incentivize providers to allocate more resources (e.g., time) to properly evaluating pain conditions [17]. Hence, they could prompt providers to prioritize opioid medications over non-opioid or non-pharmacologic treatments [18,19,20,21]. On the other hand, incentivizing providers may not necessarily yield the most desirable outcomes. For example, incentivizing based on patient’s satisfaction/experience of care might have propelled providers to opioid prescription [4,22,23,24]. For other instances where considering monetary incentives for providers has resulted in mixed outcomes, see [25,26].

#### 3.1.2. Practice Guidelines and Healthcare System Structures

The literature has raised three issues with existing guidelines for opioid prescription, particularly the CDC guidelines [11]:

- Providers do not have a clear idea about how to easily implement these guidelines in their practices or there exist uncertainties surrounding the impact of the recommendations on patient pain levels, particularly in the presence of comorbidities [27].
- Across different specialties/medical conditions, (i) there is no consensus among providers in selecting optimal treatments, and (ii) there are various perspectives on how opioids are deemed appropriate, resulting in many of the providers not aligning with the guidelines and/or significant variations among them in opioid prescription [28,29,30]. On a similar note, emergency departments (EDs) are shown to be more aligned with the CDC guidelines than non-EDs [31].
- Even for the same medical condition, there is substantial variation in opioid prescription among providers. This can be a direct consequence of the issue raised under the first item in this list [32,33].

Furthermore, regarding the system structure, we note the dual drug benefit use among Veterans Affairs (VA) and Medicare Part D enrollees, where about 25% of VA enrollees who use opioids are reported to also obtain opioid prescription from dual sources [34,35].

#### 3.1.3. Multi-Modal Pain Management

Non-opioid pharmacological (e.g., acetaminophen and nonsteroidal anti-inflammatory drugs) and non-pharmacological treatments (e.g., physical therapy, chiropractic, acupuncture, relaxation techniques, etc.) are not only shown to be associated with lower rates of opioid prescription and misuse [36,37,38,39], but reported to be as effective as opioids in managing pain conditions, especially chronic conditions [40,41,42]. As another medium, cannabis use or legalizing it in many U.S. states has been shown to be associated with lower rates of opioid prescriptions [43,44,45,46]. However, there is also evidence that using cannabis may not completely replace opioids in addressing pain complications [47]. That said, despite patients’ willingness to be engaged with alternative non-opioid treatments and healthcare organizations (e.g., VA) that have already started adopting such treatments [48,49], there are barriers in their uptake such as high cost, poor reimbursements to providers under payment mechanisms such as FFS, lack of coverage for some treatments such as acupuncture and psychological interventions, skepticism of patients towards these treatments once they have started taking opioids, and nonadherence to these treatments in the long term among patients with chronic pain [21,50,51,52,53].

#### 3.1.4. Initiatives for Opioid Prescription/Side Effects Reduction

Numerous efforts have been reported to reduce the prescribing of opioids and/or incidence of OUD/SUD. These include the implementation of prescription drug monitoring programs [54,55], statewide Medicaid program initiatives such as coordinated care organizations [56,57,58], educational outreach and academic detailing for providers [59,60,61], advances in medicine/surgery that lower post-surgical dependence on narcotics [62,63], the fentanyl patch-for-patch program [64], pharmacy consult intervention [65], quality measure development and/or quality improvement [66], using data analytics to predict the risk of overdose [67], and schedule change of opioid analgesics [68]. Other initiatives have been reported to be less successful in this regard; for example, the adoption of controlled substance laws, which are reported to not be associated with lowering the prescription of opioids or overdose incidence among disabled Medicare beneficiaries [69].

#### 3.1.5. Physician-Patient Shared Decision Making

In pain management, the misalignment between physicians and patients on treatment goals is reported to adversely impact pain management outcomes, mainly because patients’ first objective is typically to reduce pain intensity, while providers’ first priorities are to enhance functioning and diminish medication side effects [70]. It has also been reported that pain management quality may be associated with the quality of the physician-patient interaction, and this is impacted by factors like provider experience and knowledge, proper prioritization of discussing pain severity among provider’s activities, and providers’ past unpleasant encounters with patients [71].

### 3.2. Stakeholder Misalignment After Onset of OUD/SUD: An Intervention Perspective

When patients are at risk for or diagnosed with OUD/SUD, interventions center on harm reduction programs, including medication assisted treatment (MAT) and opioid substitution programs—both benefiting from medications such as methadone, naltrexone, buprenorphine, and naloxone, syringe access/exchange programs, and other initiatives such as screening, brief intervention, and referral to treatment [72,73,74,75]. Despite their efficacy, the majority of people suffering from OUD/SUD lack access to treatments [76]. Therefore, the literature has mainly focused on barriers and facilitators to adopting these medications. We present these studies in Section 3.2.1 and Section 3.2.2.

#### 3.2.1. Barriers in Adopting Treatments

Induced by the healthcare system: The barriers include lack of full or proper insurance coverage (e.g., in the California Medicaid program, naloxone is covered as an FFS medication, and managed care plans like capitation do not cover the drug), high costs of medications, limited number of providers/counselors resulting in a dearth of programs or long waiting lists, a low percentage of licensed physicians having a secured waiver that is required to provide MAT or the majority of counties lacking a treatment-waivered physician, insufficient education among pharmacists (this can be resolved by educational materials through improved FDA-approved formulations), the short-term period of opioid substitution programs, and bureaucratic requirements for program entry/enrollment [73,77,78,79,80,81,82,83,84].

Induced by providers and patients: The barriers include competing time for providers’ limited practice time, preventing them from allocating enough time to properly evaluate patients’ risk of OUD/SUD, lack of interest in treating OUD/SUD, care fragmentation and distrust in the quality of care between PCPs and specialists, stigma surrounding the use of these treatments among patients and providers, provider’s stigma in dealing with OUD/SUD patients, being less receptive to patients’ treatment preferences (magnifying the importance of shared decision-making and physician-patient interaction), and nurses having low motivation/role support in working with patients [77,81,82,85,86,87,88]. Patients also deem transportation/mobility as a barrier in seeking treatments, especially in rural settings [88]. In addition, patients’ demographic and physiological characteristics (e.g., male gender, minority race, history of opioid overdose, and hepatitis C) are shown to be associated with a higher likelihood of abrupt discontinuation of treatments [80].

#### 3.2.2. Facilitators to Adopting Treatments

Expanding capacity of treatments and providers: Adequate monetary incentives and reimbursement for providers, reducing regulatory burdens, providers’ education, private insurance coverage, and utilizing state subsidies are reported to impact the successful recruitment of providers [89,90,91]. Real-world instances of initiatives include the Substance Abuse and Mental Health Services Administration and Health Resources and Services Administration (SAMHSA-HRSA) joint project on expanding the use of medications in safety-net settings [92], SAMHSA’s Addiction Technology Transfer Center Network [93], CVS Pharmacy providing naloxone without prescriptions in most states [94], California implementing a state-wide hub-and-spoke model to improve access to OUD treatments [95], improving the rate of follow-up treatments among Medicaid enrollees in Pennsylvania by offering incentives to providers [96], and the SUPPORTAct expanding Medicare coverage to include bundled payment for treatments [97]. By contrast, in the first three year implementation of Global Payment and Accountable Care by Blue Cross Blue Shield of Massachusetts, no significant impact on using treatments was observed [98].

Improving treatment adherence and program retention: To improve retention in treatment programs or increase the number of days of opioid/drug abstinence, initiatives have included contingency management and financial incentives for patients [99,100,101,102,103,104,105,106,107] (see [108] for a review). Challenges associated with these initiatives include the use of monetary incentives to buy drugs [109,110] and diversion or misuse of methadone and buprenorphine [111,112]. Goods-based incentives may lower such risks; however, they impose higher operational costs [113]. In addition, counselors may exhibit resistance towards contingency management and financial incentives, which could necessitate educational outreach and training [114]. As other initiatives have been demonstrated to yield positive outcomes, one can refer to syringe access/exchange programs [74,115,116] and the use of technology such as therapy observation via a mobile application [117,118,119].

Role of employers: Employment rates among drug-dependent people are far lower than average rates for the U.S. population [120]. To this end, employers are deemed effective sources for establishing reinforcement strategies for drug abstinence and treatment adherence [121]. Tools, such as employment-based behavioral reinforcement and vocational problem-solving training, are reported to positively impact employment rates, opioid abstinence, and treatment adherence among opioid-dependent workers [122,123,124,125,126]. There is also evidence that employers who have educated workers and monitored opioid use among them, expanded capacity on OUD/SUD treatments, and limited opioid availability via modified health plans have observed little to no negative impact on their productivity levels [127]. Furthermore, the roles of employers along with the government and work associations in addressing OUD/SUD and providing treatments for workers were discussed in [128].

## 4. Discussion

This review highlights the misalignment of incentives across stakeholders as an important, but, to date, often overlooked contributor to the ongoing opioid epidemic. In this review, we identified various conditions, surrounding the roles of stakeholders, that have contributed to misaligned incentives by compromising the cost, quality, or access to care in the opioid crisis. Prior to experiencing OUD/SUD (the prevention stage), misalignment typically occurs between:

- Payers, providers, and patients due to conventional payment mechanisms such as FFS and capitation, lack of proper insurance coverage for multi-modal pain management, and system structures such as dual drug benefit programs for VA and Medicare Part D enrollees resulting in care fragmentation;
- Policy makers and providers due to guidelines that are not easily translatable for implementation in practice;
- Providers and patients due to lack of shared decision making on treatments, which is also common in the intervention stage.

All of the studies characterized as prior to the development of the patient’s OUD/SUD (prevention) and related to multi-modal pain management and initiatives for opioid prescription concerned all three potential misalignment sources, i.e., cost, quality, and access. None of the five prevention categories encompassed all of the stakeholder categories identified by our search, although the majority concerned payers, providers, and patients.

After experiencing OUD/SUD (the intervention stage), misalignment typically exists between:

- Payers, providers, and patients due to lack of proper insurance coverage for OUD/SUD treatments, the limited number of providers for prescribing treatments, and lack of effective incentives and reimbursements for providers;
- Pharmaceutical companies, payers, and patients due to the high cost of medications;
- Providers (PCPs and specialists) due to care fragmentation and lack of proper guidelines to streamline pathways for patients.

In contrast to the studies categorized as the prevention phase, those related to barriers and facilitators of adopting treatments after development of the patient’s OUD/SUD (intervention phase) all involved misalignment related to cost, quality, and access.

Although we have not carried out a formal systematic literature review, we would like to point out that, overall, many of the studies, including those driving changes in practice guidelines and reimbursement or confirming/purporting disincentive mechanisms, are of a moderate quality. Furthermore, the majority of studies analyzed in this review have been published within 2010–2019 (the second half of our search timeline). Therefore, the results presented here are less impacted by some of the developments in the early 2000s (e.g., unregulated marketing strategies of pharmaceutical companies) [129]. Nevertheless, our findings support the hypothesis that misaligned incentives play a significant role in the opioid epidemic. That said, they would not solely explain the totality of the current opioid crisis, in that efforts to aligning incentives among stakeholders may not always alleviate this crisis or, while improving one aspect, could worsen another dimension in this epidemic. For example, ineffective performance-based payment mechanisms do not necessarily promote quality of care in pain management, such as those utilizing patients’ satisfaction scores or experience of care. Perhaps, this is one of the reasons that mechanisms like bundled payments are becoming more common in pain management among both CMS and private insurance companies [130]. As another example, we note that many initiatives aimed at curbing the supply of opioids have not addressed how patients in dire pain conditions have been impacted [131]. Therefore, we stipulate that any effort that fails to account for both ends of the spectrum (e.g., valuing the risk of OUD/SUD while downplaying the risk of un-/under-treated pain) will likely fail in alleviating the opioid crisis thoroughly.

In addition to the foregoing issues, there are other factors that have received little to no attention in the literature, and accounting for them in incentive mechanisms can yield more impactful outcomes:

(1) The co-occurrence of OUD/SUD and chronic pain can impose pressure on providers due to multi-layered and complex treatment requirements, lack of patient improvement for either condition, and care fragmentation caused by ineffective pain management referrals [6].
(2) Guidelines that promote curbing the supply of opioids may have unintended consequences such as the increase in the number of deaths caused by fentanyl misuse. In the presence of conflicting interests, one can investigate how facilitating aligning incentives can contribute to remedying such effects.
(3) Stigma and discrimination against people with concurrent OUD/SUD and mental health disorders can stymie an effective care delivery process [132].
(4) Although incurred medical expenditures for OUD/SUD would be higher than that for under-treated pain [133], employers’ cost of lost productivity would not be much different, because their employees could miss work due to both OUD/SUD and unrelieved pain [134,135]. Hence, the role of employers should not be limited to expanding access to OUD/SUD treatments. Indeed, employers’ contribution to employment-based insurance coverage would impact the availability of treatment options and the cost of prescription drugs [136], which, in turn, affects pain management outcomes.
(5) Strategies like contingency management, aimed at improving OUD/SUD treatment adherence and retention in opioid substitution programs, have been reported to be effective only in the short term (due to financial/resource limitations), and their efficacy over the long term is yet to be investigated [137].
(6) The timing of initiating OUD/SUD treatments is a deciding factor in their success. However, patients at higher risk may not be always easy to identify. To address this, one can benefit from points of access to patients to potentially initiating treatments. These include ED visit/hospital admission [138,139,140,141,142] and incarceration [143,144,145,146,147,148,149]. Employing techniques like screening, brief intervention, and referral to treatment (SBIRT) can also be helpful in this regard [75].
(7) Behaviors like opioid injections can increase the risks of HIV and hepatitis C virus (HCV) infection. The co-occurrence of these conditions could make patients more vulnerable against the risks of OUD/SUD, and hence, extra care should be taken when dealing with such instances [150,151,152,153,154].
(8) As a result of opioid consumption ramping up during the COVID-19 pandemic [9,10], the long-term rates of OUD/SUD can be impacted as well, which can inevitably aggravate misaligned incentives. In addition to the avenues discussed thus far, this is another stream that warrants further investigation and knowledge production.

Our review has some limitations. First, even though we cover a wide range of topics associated with misaligned incentives, our method for including articles may prevent us from generalizing our findings across the whole opioid epidemic. Second, we did not conduct a systematic literature review, and hence, caution should be exercised with respect to the quality of reviewed articles or recommendations made with respect to mechanisms that trigger misaligned incentives among stakeholders. Third, in many of the studies reviewed, we construed the notion of misaligned incentives by exploring evidence on the main driving factor behind this misalignment: triple aims (cost, quality, or access) being compromised in the epidemic. This may be related to lack of empirical evidence where such misalignments are brought further to the center of attention. This could warrant investigations by conducting survey analyses among all parties involved (e.g., payers, providers, patients, etc.). Fourth, although our objective in this paper was to review the literature for evidence of misalignment, we did not quantify this notion (e.g.: How much misalignment is acceptable?). This is another avenue that is worth investigation for future research.

## 5. Conclusions

Our review sheds light on a body of literature suggesting several factors that can stir misaligned incentives between various stakeholders in the opioid epidemic. We further summarize these factors by whether or not a patient has been diagnosed with OUD/SUD yet. Along with potential challenges, we also address opportunities and strategies that have been shown to be successful in contributing to mitigating the epidemic. Of note, gaps still exist in thoroughly understanding how aligning incentives between stakeholders can help in mitigating this epidemic at full capacity. Future research is needed to further explore the impacts of factors discussed in this review from multiple, and perhaps conflicting, levels (e.g., risk of OUD/SUD versus risk of under-treated pain or risk of using opioids versus that of using fentanyl). To this end, we provided a few research directions that could be worth further investigation and knowledge production.

## Figures and Tables

**Table 1:** Summary of studies reviewed with types of stakeholders and misalignments (✓: addressed). OUD/SUD, opioid/substance use disorder.

Stage	Topic	Studies ${}^{†}$	Stakeholders ${}^{‡}$					Misalignment Source		
			PY	PR	PT	PM	OT	Cost	Quality	Access
Prevention	Payment Mechanisms, Reimbursement Schemes, and Incentives	[4,15,16,17,18,19,20,21,22,23,24,25,26] (13: 2010–2019)	✓	✓	✓	—	—	✓	✓	—
	Practice Guidelines and Healthcare System Structures	[27,28,29,30,31,32,33,34,35] (9: 2010–2019)	—	✓	✓	✓	—	—	✓	✓
	Multi-Modal Pain Management	[21,36,37,38,39,40,41,42,43,44,45,46,47,48,49,50,51,52,53] (1: 2000–2009, 18: 2010–2019)	✓	✓	✓	—	—	✓	✓	✓
	Initiatives for Opioid Prescription/Side Effects Reduction	[54,55,56,57,58,59,60,61,62,63,64,65,66,67,68,69] (16: 2010–2019)	✓	✓	✓	—	—	✓	✓	✓
	Physician-Patient Shared Decision Making	[70,71] (2: 2010–2019)	—	✓	✓	—	—	✓	✓	—
Intervention	Barriers in Adopting OUD/SUD Treatments	[72,73,74,75,76,77,78,79,80,81,82,83,84] (2: 2000–2009, 11: 2010–2019)	✓	✓	✓	—	✓	✓	✓	✓
		[77,80,81,82,85,86,87,88] (2: 2000–2009, 6: 2010–2019)	—	✓	✓	—	—	✓	✓	✓
	Facilitators to Adopting OUD/SUD Treatments	[89,90,91,92,93,94,95,96,97,98] (10: 2010–2019)	✓	✓	✓	✓	—	✓	✓	✓
		[74,99,100,101,102,103,104,105,106,107,108,109,110,111,112,113,114,115,116,117,118,119] (6: 2000–2009, 16: 2010–2019)	✓	✓	✓	—	—	✓	✓	✓
		[120,121,122,123,124,125,126,127,128] (3: 2000–2009, 6: 2010–2019)	—	—	✓	✓	✓	✓	✓	✓

${}^{†}$ For each topic, numbers in parentheses represent the total number of studies published within 2000–2009 or 2010–2019. ${}^{‡}$ PY: payer/societal planner; PR: provider; PT: patient; PM: policy/guideline maker; OT: others (pharmaceutical companies or employers).

**Table 2:** Glossary of terms used in this review.

Term	Description
Stakeholder	An entity who plays a role in navigating a healthcare-related problem, e.g., payer, provider, patient, employer, pharmaceutical company, etc.
Incentive	An interest for a stakeholder, e.g., monetary (revenue), health-related (quality of life), political (implications of a proposed healthcare bill), organizational (e.g., integrity and power issues), or behavioral (e.g., psychological factors).
Misalignment	A condition caused by competing and/or conflicting interests between two or more stakeholders resulting in either an increase in the cost of care, a reduction in the quality of care, or less access to care.
Alignment	A condition where devising mechanisms among stakeholders can either lower the cost, improve the quality, or enhance the access to care. This is a relative notion in that a “complete” alignment may not be attainable in reality.
Fee-for-service	A payment mechanism where a provider is separately reimbursed for every service delivered to a patient.
Capitation	A payment mechanism where a provider is reimbursed per patient per time period.
Pay-for-performance	The general class of payment mechanisms where the provider(s) is reimbursed based on the quality of care delivered to patients. Some examples include “bundled payment” and “accountable care”.
Bundled payment	A payment mechanism where a bundled payment is paid to a group of providers per patients per episode of care.
Accountable care	A payment mechanism where a group of providers shares benefits/savings (upon high-quality delivery of care) or is penalized in reimbursements otherwise.
Managed care	Health insurance plans that provide care for enrollees at lowered cost. Different types include health maintenance organizations, preferred provider organizations, and point of service.
Care fragmentation	Care that is delivered to a patient via multiple providers while there is little to no coordination between providers.
